# Quantification of Upper Limb Movements in Patients with Hereditary or Idiopathic Ataxia

**DOI:** 10.1007/s12311-022-01485-2

**Published:** 2022-10-21

**Authors:** Joonas Lipponen, Aleksei Tiulpin, Kari Majamaa, Harri Rusanen

**Affiliations:** 1 Research Unit of Clinical Neuroscience, University of Oulu, P.O. Box 5000, 90014 Oulu, Finland; 2 Medical Research Center Oulu, University of Oulu and Oulu University Hospital, Oulu, Finland; 3 Department of Neurology, Oulu University Hospital, Oulu, Finland; 4 Physics and Technology, Research Unit of Medical Imaging, University of Oulu, Oulu, Finland; 5 Ailean Technologies Oy, Oulu, Finland; 6 Department of Electrical Engineering, KU Leuven, Louvain, Belgium

**Keywords:** Accelerometer, Ataxia, Clinical scale, Quantification

## Abstract

Assessment of ataxic movements is usually based on clinical judgment. Technical devices can be employed in the quantification of ataxic movements in addition to clinical evaluation. The effect of maximal speed in upper limb movements in ataxia patients has not been quantified. The aim was to quantify upper limb movements in patients with hereditary or idiopathic ataxia and to find features of movement that are characteristic for ataxia. We examined 19 patients with degenerative ataxia and 21 healthy controls. An ad hoc system comprising a touch screen, an accelerometer, and a gyroscope was used to measure speed, angular acceleration, consistency, and accuracy of upper limb movements. The movements were quantified during finger-to-nose test that the patients were asked to perform at their own pace and as fast as possible. Disease severity was estimated by using the Scale for the Assessment and Rating of Ataxia (SARA). The mean SARA score of the patients was 13.5. Compared to the controls the performance of the patients was slow (*p* < 0.001) and arrhythmic (*p* < 0.001), but end-point accuracy on the touch screen was intact. The SARA score correlated with the standard deviation of amplitude of angular acceleration in Z-axis (*F*(1,17) = 15.00, *p* < 0.001 with *R*^2^ = 0.47). Upper limb movements of the patients with degenerative ataxia were slower and more arrhythmic than those in the controls. The patients retained spatial end-point accuracy.

**Supplementary Information:**

The online version contains supplementary material available at 10.1007/s12311-022-01485-2.

## Introduction

Assessment of severity in ataxic disorders is usually based on an expert opinion or determined by using clinical semiquantitative rating scales, such as the Scale for the Assessment and Rating of Ataxia (SARA) [1]. Although inter-rater reliability and test–retest reliability of these grading systems are reportedly high, they are subjective in nature and do not produce metric variables [2]. The hallmarks of ataxic limb movements are temporospatial errors in the form of end-point inaccuracy and timing lapses, increased variability in repeated movements, slowness, and altered trajectories [3]. Evaluation of these characteristics is based on the clinician’s judgment rather than any objective measure. Moreover, the complicated nature of multi-joint movements necessitates sophisticated adjunct methods that help clinicians to detect minute changes in the performance of these actions and to truly evaluate the different components of ataxic movements [3–5]. In SARA, the evaluation of finger-to-nose test (FNT) is based on the severity of kinetic tremor. Therefore, it is not optimal for assessing individual components of ataxic movements, such as altered trajectories or difficulties in initiation and termination of movements.

It has been proposed that limb dysmetria is speed dependent and that speed of movement is decreased in ataxia patients presumably as an adaptive mechanism [3]. To assess the speed dependency of limb dysmetria, devices that produce objective data are needed. Accelerometers and other quantitative methods have been used to assess movement in ataxia patients [4–18]. Interestingly, temporal errors of ataxic movements have been repeatedly detected, while spatial end-point accuracy varies between studies [8–13, 17, 18]. To the best of our knowledge, the effect of maximal speed on a multi-joint movement in an ataxia patient has only been studied in walking [14, 15].

In this study, we set out to search for a novel method for the quantification of upper limb ataxia that would reliably detect characteristic features of ataxic movement and that would correlate with the clinical SARA score. In addition, we aimed to quantify the effect of maximal speed on a goal-oriented multi-joint upper limb movement.

## Materials and Methods

### Subjects

We enrolled 21 healthy controls (10 men) and 19 adult ataxia patients (nine men) from the cohort that we have previously ascertained [19]. Nine patients had a genetic ataxia including two patients with m.8561C > G in *MT-ATP6/8* [20], one patient with autosomal recessive spastic ataxia of Charlevoix-Saguenay [21], one patient with fragile X tremor ataxia syndrome (FXTAS), one patient with pontocerebellar hypoplasia type 6 (PCH6), one patient with homozygous p.W748S in *POLG1* causing mitochondrial recessive ataxia syndrome (MIRAS), one patient with ataxia teleangiectasia (AT), and two patients with a biallelic AAGGG_exp_ in *RFC1* causing cerebellar ataxia, neuropathy, and vestibular areflexia syndrome (CANVAS) (Table 1). The phenotype and the severity of disease varied between the subjects. All patients were clinically evaluated by an experienced neurologist (HR). Assessment of ataxia using SARA and measurements using accelerometer were performed on the same day.

**Table 1:** Demographics of the ataxia patients

Patient	Age (years)	Sex	Age of onset (years)	Diagnosis	SARA score
P1	68	F	58	IA	2
P2	68	M	58	IA	4.5
P3	62	M	57	FXTAS	5
P4	50	M	43	IA	8
P5	27	F	2	PCH6	9
P6	63	F	52	CANVAS	10.5
P7	59	F	50	IA	10.5
P8	57	M	45	CANVAS	12
P9	41	M	24	MIRAS	12
P10	72	M	60	IA	13.5
P11	57	F	20	MA	15
P12	82	F	68	IA	15.5
P13	84	M	70	AT	17
P14	82	F	75	IA	18
P15	58	F	20	ARSACS	19
P16	62	M	20	MA	20
P17	49	M	14	IA	20
P18	22	F	2	IA	20
P19	65	F	55	IA	24.5

*ARSACS*, autosomal recessive spastic ataxia of Charlevoix-Saguenay; *AT*, ataxia teleangiectasia; *CANVAS*, cerebellar ataxia, neuropathy, vestibular areflexia syndrome; *F*, female; *FXTAS*, fragile X-associated tremor/ataxia syndrome; *IA*, idiopathic ataxia; *M*, male; *MA*, mitochondrial ataxia (m.8561C > G in *MT-ATP6/8*); *MIRAS*, mitochondrial recessive ataxia syndrome; *PCH6*, pontocerebellar hypoplasia type 6; *SARA score*, total SARA score

The control subjects did not have neurological, musculoskeletal, or other disorders that would impair their ability to perform FNT nor did they use medications that would affect their performance.

### Measurement Setup

**Fig. 1:**
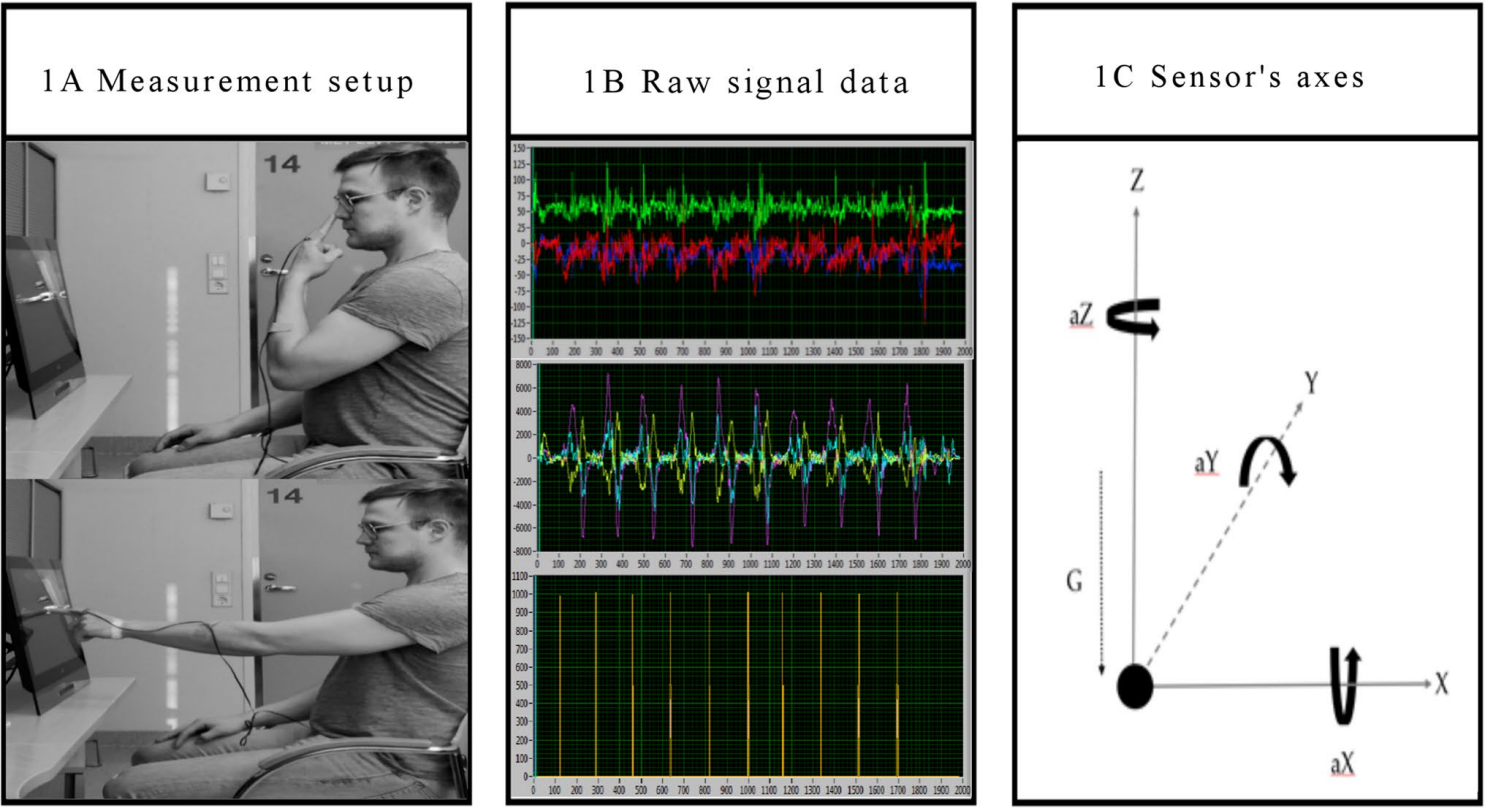
**A** Measurement setup. The sensor was attached to the proximal phalanx of the II digit in the subject’s dominant hand. There was a 1 cm target dot displayed on the screen. Subject would alternate between the dot and their nose. **B** Raw signal data of the accelerometer (top), gyroscope (middle), and touch screen (bottom) taken from a healthy subject’s measurement. Individual colors represent different axes of acceleration, angular acceleration, and touch screen coordinates as follows: red = acceleration in X-axis, blue = acceleration in Y-axis, green = acceleration in Z-axis, light yellow = angular acceleration in X-axis, light blue = angular acceleration in Y-axis, violet = angular acceleration in Z-axis, amber = horizontal touch screen coordinate, rose = vertical touch screen coordinate

The instruments comprised of a lightweight triaxial accelerometer (LIS302DL, LSM303D, STMicroelectronics, Schiphol, Netherlands) and triaxial gyroscope (L3G4200D, L3GD20, STMicroelectronics) a laptop (K53E-SX1849V, ASUS, Taipei, Taiwan) and a touch screen (T232HL, Acer, New Taipei, Taiwan). The setup and software were manufactured by IProtoXi (IProtoXi Oy, Oulu, Finland). Two types of sensors were used, one with a sampling rate of 100 Hz and 8-bit resolution and the other with a sampling rate of 95 Hz and 16-bit resolution. The range of detection in both sensors was ± 2 G for the accelerometer and ± 250 degrees per second for the gyroscope (Fig. 1).

The data from the sensors were transmitted to the laptop via Bluetooth. The sensor of the accelerometer was attached on the dorsum of the proximal phalanx of the second digit in the subject’s dominant hand. The Bluetooth transmitter was placed in the patient’s pocket and the wire from the sensor to the transmitter was secured so that it did not hinder hand movements. The software system was developed using LabView (v.2010).

### Finger-to-Nose Test

Test subject sat in front of the touch screen so that they could easily reach the center of the screen without tilting or rotating their body towards the screen and with their elbow slightly bent at the end of the extension towards the screen (Fig. 1). There was an immobile dot displayed on the touch screen. Before the test started the subject was asked to place his/her hand on the thigh. When the test started the subject would tap the dot on the screen and then the tip of the nose with the index finger at his/her own pace (FNT slow). The subject would alternate between the dot and the nose, and one attempt was intended to consist of at least 10 dot-to-nose rounds. The purpose of FNT slow was to evaluate the accuracy, rhythmicity, timing, and stability of a goal-oriented multi-joint upper limb movement performed at subject’s preferred speed. FNT slow was performed three times, and before each attempt, the subject was verbally motivated to concentrate on being as accurate as possible.

After the three attempts of FNT slow, the subject was asked to perform finger-to-nose test as fast as possible (FNT fast) in order to evaluate the accuracy, rhythmicity, timing, and stability of the movement performed at subject’s maximal speed. This test was performed three times, and before each attempt, the subject was verbally motivated to perform the test as fast as possible.

It was required that an accepted attempt in FNT contained no unintended hits or finger dragging on the touch screen. The hit was deemed unintentional, if more than one consecutive hits were recorded on the screen and if there was no accelerometer signal showing movement towards the nose between the hits. Finally, all accepted attempts were inspected, and the one with least artifacts was used. In FNT fast, the fastest attempt defined by the number of intentional hits on the touch screen was selected for the analysis (Table 2).

**Table 2:** Number of accepted attempts in FNT and number of dot-to-nose rounds in the 10-s sample used in the analysis

Subject	FNT slow		FNT fast	
	Attempts (*N*)	Rounds (*N*)	Attempts (*N*)	Rounds (*N*)
P1	1	8	3	10
P2	2	4	2	10
P3	1	7	2	10
P4	2	4	2	7
P5	1	4	1	6
P6	1	5	1	6
P7	1	5	2	7
P8	2	6	1	7
P9	2	10	2	9
P10	1	4	3	7
P11	2	6	3	5
P12	2	6	2	8
P13	2	3	1	4
P14	2	2	1	4
P15	1	6	2	9
P16	2	5	2	11
P17	2	2	2	5
P18	2	4	2	4
P19	2	4	2	5
C1	2	7	2	11
C2	2	8	2	11
C3	2	9	1	11
C4	2	10	1	12
C5	2	7	2	9
C6	1	5	3	10
C7	1	10	1	11
C8	3	7	2	10
C9	2	7	3	10
C10	1	6	3	10
C11	2	5	2	11
C12	2	7	2	11
C13	2	8	2	11
C14	1	10	2	13
C15	1	6	2	8
C16	1	8	1	12
C17	2	5	1	13
C18	3	6	2	10
C19	3	6	2	9
C20	1	6	2	9
C21	2	6	2	10
Mean, P	1.63	5.00	1.89	7.05
Mean, C	1.81	7.10	1.90	10.57

*P*, patient; *C*, control; rounds = dot-to-nose rounds in the 10-s sample. Attempts = accepted attempts. Patients P14 and P17 were excluded from the analysis of FNT slow, because the 10-s sample contained only two dot-to-nose rounds

### Scale for the Assessment and Rating of Ataxia

SARA comprises of eight items including gait, stance, sitting, speech, finger chase (FC), finger-to-nose test, fast alternating hand movements (DDK), and heel-shin slide (HSS). The scale has a maximum score of 40. Gait is scored 0–8, stance 0–6, sitting 0–4, speech 0–6, and the limb tests 0–4. Right and left side are scored separately for the limb tests (FC, FNT, DDK, HSS) and the mean is used in the total score [1].

### Data Pre-processing

The accelerometer measured the difference between acceleration of the sensor and the gravity in horizontal direction (X-axis), in anterior–posterior direction (Y-axis), and in vertical direction (Z-axis).

**Fig. 2:**
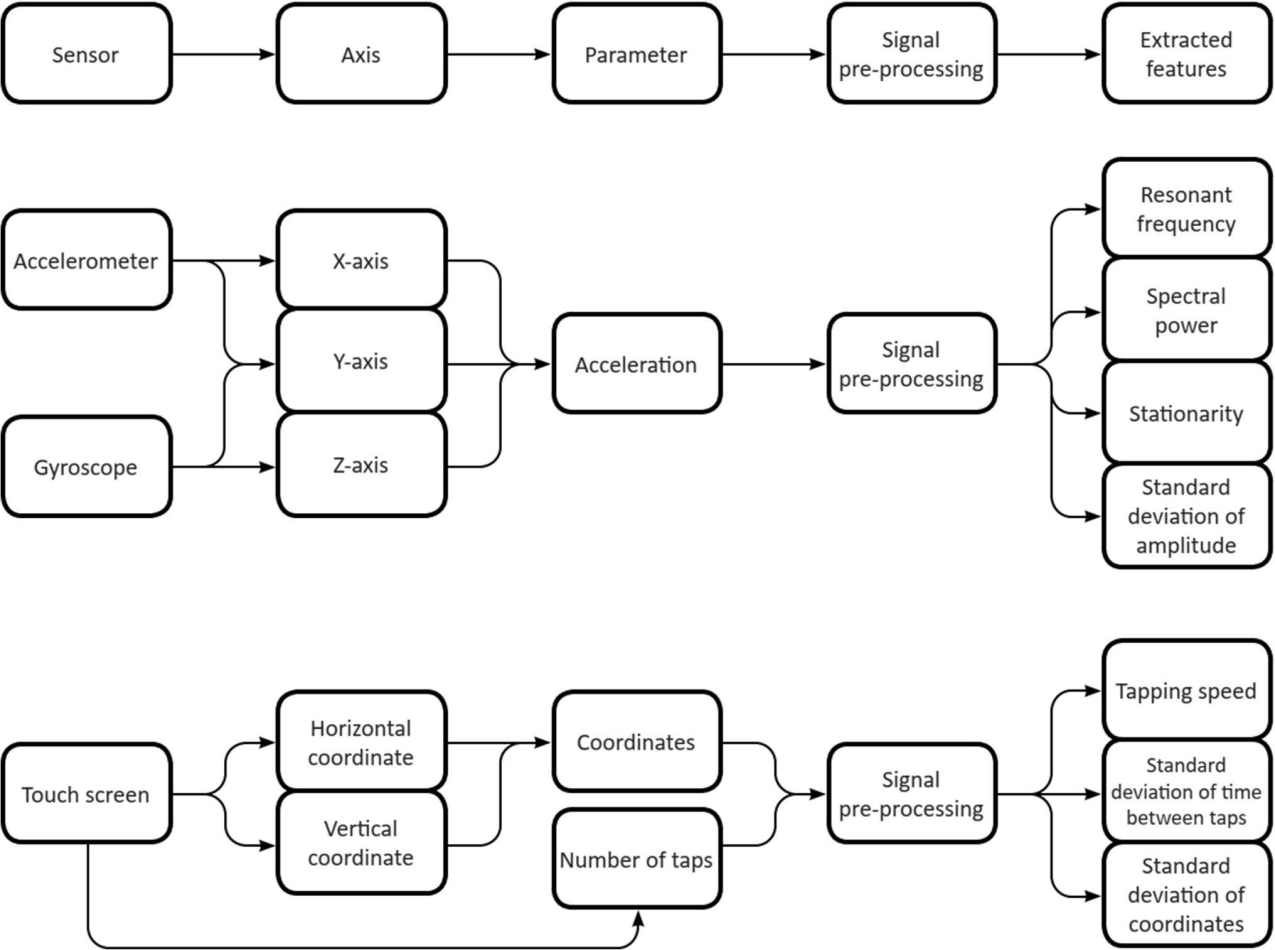
Flowchart detailing the process of feature extraction from the touch screen, accelerometer, and gyroscope signals. Resonant frequency, spectral power, stationarity, and standard deviation of amplitude were extracted separately for each axis yielding a total of 24 variables from the gyroscope and accelerometer signals

The signals measured from the sensors were quantified as follows. Firstly, we filtered the signals within 1–15 Hz using a Butterworth filter of 6th order to remove high-frequency noise. Secondly, artifacts in the beginning and the end of the signal were removed by cropping each signal to a 10-s sample with a center in the middle timestamp of the acquired signal. The sample included a sufficient number of dot-to-nose rounds to be used in the analysis (Table 2). Finally, we removed the zero-frequency trend by subtracting the mean amplitude of the signal (Fig. 2).

### Quantitative Parameters

We aimed to quantify the movements in terms of resonant frequency (RF), stationarity (STA), and amplitude, as well as the consistency, accuracy, and speed in screen tapping. Tapping speed was defined as the number of screen taps in 10 s. Coefficient of variation (CV) was used to quantify temporal variation of the movement. Temporal CV was calculated by dividing the standard deviation of time between taps by the mean of time between taps. The total amplitude of the movements was quantified by signal power (SP) that was computed as the sum of squared values of the signal divided by the number of samples. Standard deviation of the amplitude of acceleration (SDA) was used to quantify the variability of the acceleration. The dominant frequency was computed using the Fourier transform by identifying a frequency corresponding to the maximum in the signal spectrum. Spatial end-point accuracy was defined as the standard deviation of all the X–Y coordinates produced by the taps on the touch screen and normalized by screen resolution. In total, 27 parameters were extracted (Fig. 2, Supplementary Table 1). We used Numpy [22] software library for the Python programming language (v.3.6) to develop the codes for data processing.

### Statistical Analysis

Statistical analysis was carried out using SPSS Statistics 25 (IBM, Chicago, IL, U.S.A.). For each variable, the assumption of normal distribution was assessed by the Kolmogorov–Smirnov test. To assess differences in the extracted variables of FNT or demographic variables between groups, an independent sample *t*-test or Mann–Whitney *U* test was performed as appropriate. A Bonferroni correction was used when multiple tests were performed. A simple linear regression was used to predict SARA score based on accelerometer data. Correlations between the variables and SARA score were evaluated using Pearson’s correlation coefficient or Spearman’s rank correlation coefficient. In order to study correlations between temporal and spatial variability, both temporal CV and spatial variables were evaluated with Pearson’s correlation coefficient or Spearman’s rank correlation coefficient. Correlation coefficients >|0.5| were deemed significant. After a Bonferroni post hoc correction, the level of significance was set at *p* < 0.002. Accelerometer, gyroscope, and touch screen features with the highest significant correlation coefficients were used in a linear regression analysis. All the assumptions for linear regression were met. Cross-validation with an 80% sample was used to create predicted SARA scores. The level of significance for the regression was set at *p* < 0.05. A paired-samples *t*-test or Wilcoxon signed-rank test was performed as appropriate to evaluate the effect of speed on total movement amplitude, standard deviation of amplitude of acceleration, resonant frequency, stationarity, tapping accuracy, and temporal variability within groups.

## Results

### Subject Demographics

The age of the patients was 59.4 ± 16.6 years (mean ± standard deviation; range, 22–84 years) and the mean age at disease onset was 41.7 ± 22.1 years (range, 2–75 years). The median disease duration was 14 years (range, 5–42 years) (Table 1) and the mean SARA score was 13.5 ± 6.1. SARA items measuring upper limb functions gave mean subscore 1.5 (range, 0–4) for DDK, 1.1 (range, 0–3) for FNT, and 0.8 (range, 0–2) for FC. The mean age of the controls was 53.7 ± 18.3 years (range, 24–93 years). There were no significant differences in the age or sex distribution between the patients and controls.

### Quantitative Analysis of Upper Limb Movements

**Fig. 3:**
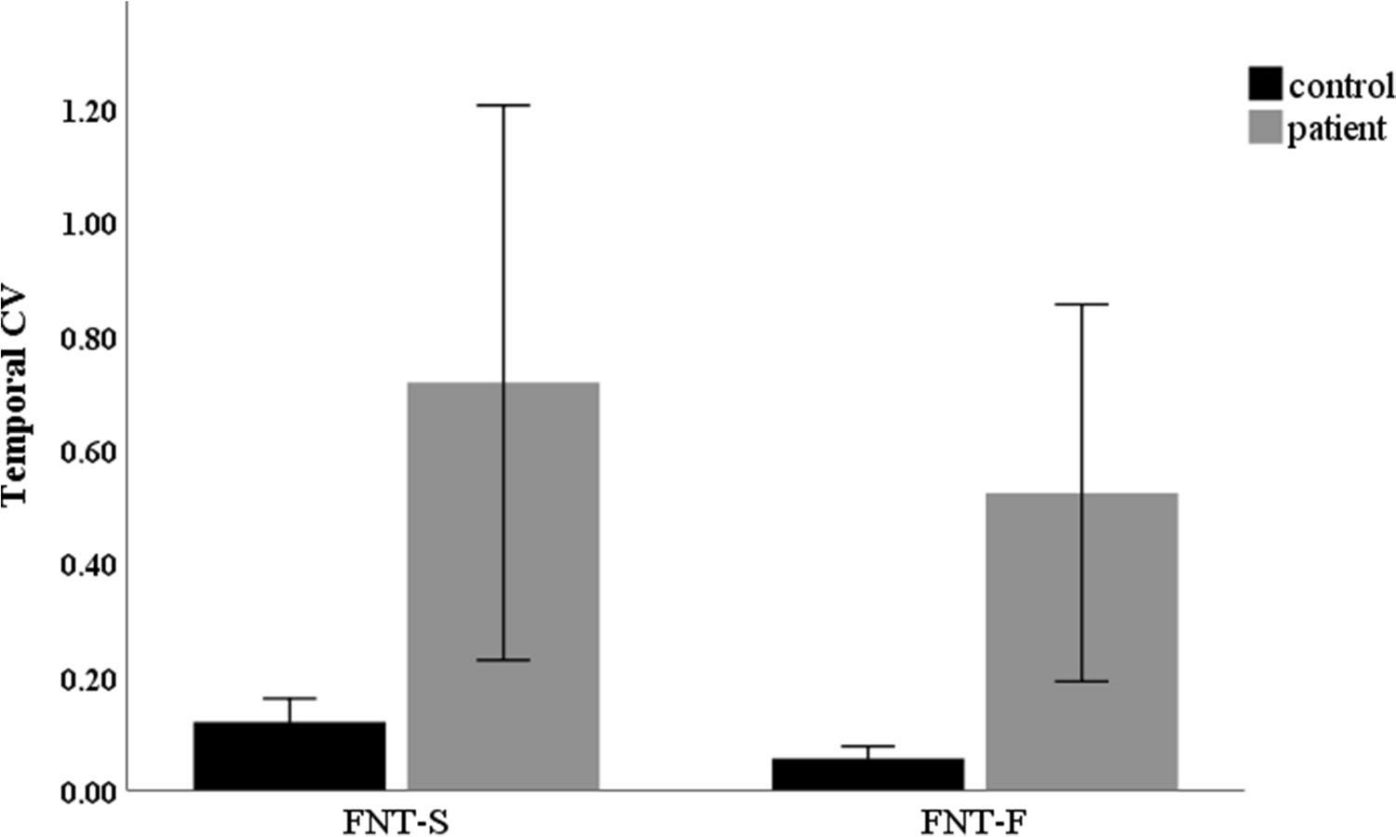
Temporal variation of movement in FNT. A trend towards temporal invariance was visible in both groups but was not significant after the Bonferroni post hoc correction (controls *Z* =  − 2.76, *p* = 0.006; patients Z =  − 0.36, *p* = 0.723). Error bars represent 95% confidence intervals

Preferred tapping speed and maximum tapping speed were significantly slower in the patients than in the controls (Table 3). Interestingly, the ratio of preferred to maximum tapping speed was similar being 0.65 for controls and 0.70 for patients (*p* = 0.37 for difference). Coefficient of variation (CV) of tapping speed was used to evaluate the timing of movements. CV of tapping speed was higher in patients than in controls in both FNT slow and FNT fast (Table 3) being compatible with higher arrhythmicity in the upper limb movements of the patients. Interestingly, CV of tapping speed was slightly lower both in the patients and the controls, when FNT was performed at a maximal speed (Fig. 3).

**Table 3:** Temporal variability of movement in FNT

Test		Controls	Patients	*p*-value
FNT slow	Tapping speed (1/s)	0.71 (0.50–1.00)	0.50 (0.30–1.00)	0.002
	SD of time between taps (s)	0.05 (0.01–0.31)	0.10 (0.01–1.11)	0.069
	CV of TS	0.09 (0.02–0.30)	0.27 (0.03–3.71)	0.001
FNT fast	Tapping speed (1/s)	1.10 ± 0.13	0.71 ± 0.23	< 0.001
	SD of time between taps (s)	0.03 (0.02–0.23)	0.10 (0.03–0.97)	< 0.001
	CV of TS	0.03 (0.02–0.21)	0.24 (0.04–2.42)	< 0.001

*TS*, tapping speed; *SD*, standard deviation; *CV*, coefficient of variation. The values are means ± SD or medians (range) as appropriate. Patients P14 and P17 with only two dot-to-nose rounds in FNT slow were excluded from the analysis

Spatial end-point accuracy in FNT slow or FNT fast did not differ between the patients and controls (Table 4). Moreover, there was no significant decrease in spatial end-point accuracy among the patients in FNT fast compared to FNT slow (Supplementary table 2.).

**Table 4:** Spatial variables of movement in FNT

Test		Controls	Patients	*p*-value
FNT slow	Acc	0.12 ± 0.40	0.12 ± 0.07	0.937
	SDA of X	0.33 ± 0.08	0.23 ± 0.07	< 0.001^a^
	SDA of Y	0.20 ± 0.06	0.15 ± 0.05	0.020
	SDA of Z	0.25 ± 0.07	0.17 ± 0.06	< 0.001^a^
	SDA of aX	7.35 ± 2.83	4.78 ± 1.95	0.002
	SDA of aY	10.3 ± 4.26	7.64 ± 3.13	0.070
	SDA of aZ	12.0 ± 4.49	7.62 ± 3.62	0.002
	SP of X	7.51 ± 1.99	5.20 ± 1.67	< 0.001^a^
	SP of Y	4.60 ± 1.55	3.43 ± 1.20	0.190
	SP of Z	6.32 ± 1.77	3.95 ± 1.33	< 0.001^a^
	SP of aX	159 ± 63.5	109 ± 45.6	0.005
	SP of aY	188 ± 54.3	155 ± 61.8	0.009
	SP of aZ	196 ± 68.7	131 ± 56.8	0.003
FNT fast	Acc	0.10 ± 0.03	0.11 ± 0.06	0.963
	SDA of X	0.63 ± 0.23	0.34 ± 0.14	< 0.001^a^
	SDA of Y	0.37 ± 0.17	0.22 ± 0.08	0.001^a^
	SDA of Z	0.46 ± 0.14	0.25 ± 0.10	< 0.001^a^
	SDA of aX	13.5 ± 4.26	7.19 ± 0.74	< 0.001^a^
	SDA of aY	20.1 ± 1.70	10.7 ± 1.28	< 0.001^a^
	SDA of aZ	25.7 ± 2.08	12.4 ± 1.87	< 0.001^a^
	SP of X	13.8 ± 4.22	7.79 ± 2.80	< 0.001^a^
	SP of Y	8.91 ± 2.97	5.38 ± 2.11	< 0.001^a^
	SP of Z	11.0 ± 3.22	6.02 ± 2.47	< 0.001^a^
	SP of aX	311 ± 96.1	170 ± 82.2	< 0.001^a^
	SP of aY	350 ± 125	211 ± 84.0	< 0.001^a^
	SP of aZ	397 ± 142	211 ± 109	< 0.001a

*t*-test or Mann–Whitney *U* test were used as appropriate to compare group differences. acc, spatial end-point accuracy; aX, angular acceleration in X-axis; aY, angular acceleration in Y-axis; angular acceleration in Z-axis; *SDA*, standard deviation of amplitude; *SP*, spectral power within 1–15-Hz range; X, acceleration in X-axis; Y, acceleration in Y-axis; Z, acceleration in Z-axis. ^a^Significant *p*-value

Upper limb movements in FNT were measured by using accelerometer and gyroscope. The total amplitude of movement was lower in patients than in controls in both FNT slow and FNT fast. The variability of acceleration was also lower in patients than in controls in both FNT slow and FNT fast (Table 4). No significant differences in resonant frequency or stationarity of upper limb movements were found (Supplementary table 3).

### Correlation of Upper Limb Movements to Disease Severity

**Fig. 4:**
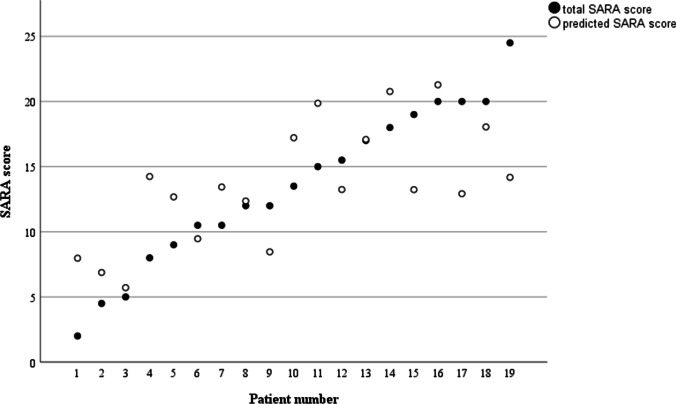
Correlation between predicted and actual SARA score. The actual SARA scores and SARA scores predicted by the variability of angular acceleration in Z-axis in FNT slow showed strong correlation (*r* = 0.69, *p* = 0.001)

Disease severity was estimated by means of the SARA score. There was a correlation, although not significant, between the SARA score and disease duration. In addition, we found that variability of angular acceleration in Z-axis in FNT slow was the only variable with significant correlation to total SARA score (*p* = 0.001, after the Bonferroni correction) (Supplementary table 4) and it was selected as the predictor variable in linear regression analysis. Cross-validation with 15 patients in the training set was performed. The predicted SARA scores and true SARA scores showed strong correlation (*r* = 0.69, *p* = 0.001, Fig. 4). SARA score could be predicted by the variability of angular acceleration in Z-axis in FNT slow ((*F*(1,15) = 9.84, *B* =  − 1.27, *p* = 0.007) with *R*^2^ = 0.41). Variables extracted from FNT fast did not correlate with SARA score. A significant regression for FNT fast was not found.

## Discussion

We quantified upper limb movements in patients with hereditary or idiopathic ataxia and found that they had slower tapping speed, but their end-point accuracy was similar to that in controls. In addition, arrhythmicity was more pronounced in the upper limb movements of the patients compared to controls as suggested by the higher CV of tapping speed.

We found that the patients performed FNT slower than the controls, but there was no difference in the end-point accuracy between the groups. In ataxia patients, goal-oriented multi-joint movements, such as FNT, become slow, less smooth, and fragmented, and have variable trajectories requiring increased adjustments [8, 10, 11]. It has been suggested that ataxia patients are able to counteract spatial end-point inaccuracy by using visual control and deceleration of the movement [8, 10, 11]. If the visual feedback of hand position or trajectory is removed, the spatial end-point inaccuracy becomes evident in ataxia patients [11]. We did not specifically examine the effect of visual control on the end-point accuracy, but our findings on the observed slowness and retained spatial end-point accuracy are in line with previous studies [8, 10, 11, 14, 15].

Fitts task is a reciprocal aiming task, in which the target size and distance are manipulated [23]. It is a test devised to examine speed-accuracy trade-off in upper limb movements. Patients with Friedreich ataxia make significantly more spatial end-point errors and have higher spatial end-point variability in Fitts task than controls [13]. In addition, temporospatial errors become more pronounced, when the target is smaller or more distant [13]. We expected to see a change in accuracy with increasing speed, but no difference in spatial end-point accuracy was observed. It has been shown that the speed of reaching motions affects the kinematics of the upper limb movement in patients with cerebellar disease. Variable joint angles and decomposition of movement become more pronounced at slow speed, while faster speed results in less decomposition but more pronounced hypermetria [16]. One reason for the lack of difference in spatial end-point accuracy might lie in the FNT itself. In FNT, the subject is supposed to hit a physical target, i.e., clinician’s finger or a dot on the touch screen. Successful hits stop the movement in anterior–posterior direction, and thus, spatial hypermetria in anterior–posterior direction cannot be assessed.

Spatial end-point accuracy seems to vary from study to study, whereas the temporal errors seem ubiquitous [8–13, 17, 18]. Dysdiadochokinesia is regarded as a cardinal sign of cerebellar disease, but it is not specific to cerebellar ataxia [24]. We did not instruct the subjects to perform FNT rhythmically. In spite of the lack of instructions, the controls performed the tasks fast and rhythmically, whereas the performance of the patients was slow and arrhythmic. Studies on the speed dependency of temporal variability of ataxic gait have revealed that variability is lowest, when ataxia patients walk at their preferred pace [14, 15]. The variability of movement increases, when ataxia patients walk slower or faster than their preferred pace [14, 15]. It has been proposed that the increased variability in fast locomotion of ataxia patients can be attributed to impairment of cerebellar pacemaker function [14, 15].

We found that variability of angular acceleration in the Z-axis, the vertical plane, in FNT slow correlated with total SARA score. Indeed, SARA score could be predicted by this variable, whereas no other significant regression equations were found for either FNT slow or FNT fast. Angular acceleration, i.e., magnitude of resonance and resonant frequency in Y-axis, gives the best correlation with the SARA score and separation between cerebellar patients and controls [5]. We agree that abnormal characteristics of angular acceleration of upper limb movements may reflect the degree of difficulty in ataxic disorders. The disagreement in the direction of the angular acceleration could be due to methodological differences. We reasoned that the sum of all the components of upper limb movements would be best reflected in the most distal part of the limb used in FNT. Therefore, we attached the sensor to the second digit and not to the wrist [5]. The effects of cerebellar disease on limb movements may vary depending on the direction of the movement. It has been proposed that the average amplitude of medial–lateral sway of straight gait is a biomarker for spinocerebellar degeneration [25]. Moreover, only sagittal features of walking show a U-shaped variability of speed dependency in patients with cerebellar disease while horizontal features do not [14]. Our study showed that ataxic disorders affect all axes of movements (Table 4). Our results on the relationship between the SARA score and variability of angular acceleration in Z-axis in a goal-oriented upper limb movement should be verified with a larger and less heterogeneous patient cohort.

FNT is used in clinical practice to evaluate upper limb ataxia. It consists of repeated exocentric goal-oriented multi-joint reaching and egocentric pointing motions, which can elucidate the core features of ataxic upper limb movements: dyssynchronous joint movements, dysdiadochokinesia, dysmetria, and temporospatial variability [24]. The cerebellum is charged with storing and/or updating internal movement models. The role of the cerebellum in the control of deft movements becomes more pronounced with increasing degrees of freedom in movement and in situations that require error correction [26]. In both SARA and International Co-operative Ataxia Rating Scale (ICARS), FNT is performed at either preferred or moderate speed [1, 27]. We agree that these semiquantitative scales should be used routinely, as they standardize the evaluation of ataxia patients, and they provide information that can be utilized to evaluate the effect of interventions and the progression of the disease. However, ataxia patients exhibit least variability of movement, when the test is performed at preferred pace [14, 15], which is why we suggest that different movement conditions, such as different speeds, should be applied in clinical evaluation to better elucidate ataxic symptoms. Movement features, such as the variability of angular acceleration in Z-axis, cannot be observed in clinical evaluation or semiquantitative scales. Objective quantitative methods should be used to develop a clinical biomarker for upper limb ataxia.

Our patients were heterogeneous in their genetic etiology and clinical phenotypes. Larger and more homogenous patient cohorts are required in order to better understand the impact of specific genotypes on ataxic upper limb movements. Quantification of movements in patients with a given genetic etiology could demonstrate differences in phenotype and enable the detection of disease-specific biomarkers [6, 9, 13, 17, 18]. Interestingly, patients with spinocerebellar ataxia type 6 may present with a phenotype, in which temporal variability in fast goal-directed single-joint movements is lower than that in the controls, whereas patients with Friedreich ataxia show significantly more frequent errors in timing, but not positional errors [18]. Low temporal variance in patients with SCA6 is associated with a more severe phenotype measured with clinical semiquantitative scales [17]. These findings highlight the need for quantitative measurements with larger number of ataxia patients with known genotypes.

Our study has some limitations. A relatively small number of dot-to-nose rounds was used for feature extraction, which may affect the results. The criteria for an accepted attempt of FNT may overestimate the spatial end-point accuracy of the patient group, as attempts with finger dragging or unintentional hits were excluded. The excluded dot-to-nose rounds were not amenable to analysis, and inclusion of the failed rounds would only add outliers or introduce unreliability into the variables. Furthermore, both controls and patients had attempts with unintentional hits or finger dragging. It is also possible that the wide age range and heterogeneity of the patients may have affected our results on end-point accuracy, although age did not correlate with any quantitative parameter extracted from upper limb movements in FNT (data not shown). Moreover, upper limb was not severely impaired in most patients, as suggested by the low SARA scores in FC, DDK, and FNT.

## Conclusions

We quantified ataxic upper limb movements using a touch screen to analyze pointing accuracy and timing. Accelerometer and gyroscope were utilized to further characterize ataxic upper limb movements and trajectories. We found that the end-point accuracy of ataxia patients did not differ from that of the controls, but their movements were slow and arrhythmic. SARA score correlated with the standard deviation of amplitude of angular acceleration in Z-axis.

### Supplementary Information

Below is the link to the electronic supplementary material.

Supplementary file1 (DOCX 38 KB)

## Data Availability

The manuscript includes all data used in this study.

## References

[1] Schmitz-Hubsch T, du Montcel S, Tezenas MD, PhD Baliko L, Berciano J, Boesch S (2006). Scale for the assessment and rating of ataxia: development of a new clinical scale. Neurology.

[2] Weyer A, Abele M, Schmitz-Hübsch T, Schoch B, Frings M, Timmann D (2007). Reliability and validity of the scale for the assessment and rating of ataxia: a study in 64 ataxia patients. Mov Disord.

[3] Manto M, Bower JM, Conforto AB, Delgado-García JM, da Guarda SNF, Gerwig M (2012). Consensus paper: roles of the cerebellum in motor control—the diversity of ideas on cerebellar involvement in movement. Cerebellum.

[4] Shirai S, Yabe I, Takahashi-Iwata I, Matsushima M, Ito YM, Takakusaki K (2019). The responsiveness of triaxial accelerometer measurement of gait ataxia is higher than that of the scale for the assessment and rating of ataxia in the early stages of spinocerebellar degeneration. Cerebellum.

[5] Krishna R, Pathirana PN, Horne M, Power L, Szmulewicz DJ (2019). Quantitative assessment of cerebellar ataxia, through automated limb functional tests. J Neuroeng Rehabilitation.

[6] Bui HT, Audet O, Mathieu J, Gagnon C, Leone M (2017). Computer-based assessment of upper-limb incoordination in autosomal recessive spastic ataxia of Charlevoix-Saguenay patients: a pilot study. J. Neurol. Sci.

[7] Matsushima A, Yoshida K, Genno H, Ikeda S (2017). Principal component analysis for ataxic gait using a triaxial accelerometer. J Neuroeng Rehabilitation.

[8] Honda T, Mitoma H, Yoshida H, Bando K, Terashi H, Taguchi T (2020). Assessment and rating of motor cerebellar ataxias with the Kinect v2 depth sensor: extending our appraisal. Front Neurol.

[9] O’Keefe JA, Bang D, Robertson EE, Biskis A, Ouyang B, Liu Y (2020). Prodromal markers of upper limb deficits in FMR1 premutation carriers and quantitative outcome measures for future clinical trials in fragile X-associated tremor/ataxia syndrome. Mov Disord Clin Pract.

[10] Menegoni F, Milano E, Trotti C, Galli M, Bigoni M, Baudo S (2009). Quantitative evaluation of functional limitation of upper limb movements in subjects affected by ataxia. Eur J Neurol.

[11] Day BL, Thompson PD, Harding AE, Marsden CD (1998). Influence of vision on upper limb reaching movements in patients with cerebellar ataxia. Brain.

[12] Gajos KZ, Reinecke K, Donovan M, Stephen CD, Hung AY, Schmahmann JD (2020). Computer mouse use captures ataxia and parkinsonism, enabling accurate measurement and detection. Mov Disord.

[13] Corben LA, Georgiou-Karistianis N, Bradshaw JL, Hocking DR, Churchyard AJ, Delatycki MB (2011). The Fitts task reveals impairments in planning and online control of movement in Friedreich ataxia: reduced cerebellar-cortico connectivity?. Neuroscience.

[14] Wuehr M, Schniepp R, Ilmberger J, Brandt T, Jahn K (2013). Speed-dependent temporospatial gait variability and long-range correlations in cerebellar ataxia. Gait Posture.

[15] Schniepp R, Wuehr M, Neuhaeusser M, Kamenova M, Dimitriadis K, Klopstock T (2012). Locomotion speed determines gait variability in cerebellar ataxia and vestibular failure. Mov Disord.

[16] Bastian AJ, Martin TA, Keating JG, Thach WT (1996). Cerebellar ataxia: abnormal control of interaction torques across multiple joints. J. Neurophysiol.

[17] Yacoubi B, Casamento-Moran A, Burciu RG, Subramony SH, Vaillancourt DE, Christou EA (2020). Temporal invariance in SCA6 is related to smaller cerebellar lobule VI and greater disease severity. J Neurosci.

[18] Corti M, Casamento-Moran A, Delmas S, Bracksieck S, Bowman J, Meyer B (2020). Temporal but not spatial dysmetria relates to disease severity in FA. J Neurophysiol.

[19] Lipponen J, Helisalmi S, Raivo J, Siitonen A, Doi H, Rusanen H (2021). Molecular epidemiology of hereditary ataxia in Finland. BMC Neurol.

[20] Kytövuori L, Lipponen J, Rusanen H, Komulainen T, Martikainen MH, Majamaa K (2016). A novel mutation m 8561C>G in MT ATP6/8 causing a mitochondrial syndrome with ataxia peripheral neuropathy diabetes mellitus and hypergonadotropic hypogonadism. J Neurol.

[21] Palmio J, Kärppä M, Baumann P, Penttilä S, Moilanen J, Udd B (2016). Novel compound heterozygous mutation in SACS gene leads to a milder autosomal recessive spastic ataxia of Charlevoix-Saguenay, ARSACS, in a Finnish family. Clin Case Rep.

[22] Harris CR, Millman KJ, van der Walt SJ, Gommers R, Virtanen P, Cournapeau D (2020). Array programming with NumPy. Nature.

[23] Fitts PM (1954). The information capacity of the human motor system in controlling the amplitude of movement. J Exp Psychol.

[24] Bodranghien F, Bastian A, Casali C, Hallett M, Louis ED, Manto M (2016). Consensus paper: revisiting the symptoms and signs of cerebellar syndrome. Cerebellum.

[25] Shirai S, Yabe I, Matsushima M, Ito YM, Yoneyama M, Sasaki H (2015). Quantitative evaluation of gait ataxia by accelerometers. J Neurol Sci.

[26] Charles SK, Okamura AM, Bastian AJ (2013). Does a basic deficit in force control underlie cerebellar ataxia?. J Neurophysiol.

[27] Trouillas P, Takayanagi T, Hallett M, Currier RD, Subramony SH, Wessel K (1997). International Cooperative Ataxia Rating Scale for pharmacological assessment of the cerebellar syndrome. J Neurol Sci.

