# Supplementary material for: Quantification of Upper Limb Movements in Patients with Hereditary or Idiopathic Ataxia
Source: Cerebellum. 2022 Oct 21;22(6):1182–91. doi: 10.1007/s12311-022-01485-2 (PMC10657283; doi:10.1007/s12311-022-01485-2)
Supplement: Supplementary file 1 — Supplementary file1 (DOCX 38 KB) [file 12311_2022_1485_MOESM1_ESM.docx]

**Supplementary table 1**. Variables used in the characterization of upper limb movements

| Variable | Explanation |
| --- | --- |
| Taps | Number of taps registered on the touch screen |
| Spectral power* | Power with 1-15 Hz spectral range in a given axis |
| Resonant frequency* | Dominant frequency of the signal in a given axis |
| Stationarity* | Stationarity of the signal in a given axis |
| Standard deviation of amplitude* | Standard deviation of the signal’s amplitude in a given axis |
| Consistency | Standard deviation of time between taps |
| Accuracy | Standard deviation of all the X-Y coordinates produced by the taps on the touch screen and normalized by screen resolution |

* Accelerometer and gyroscope data extracted individually for X, Y and Z axes

**Supplementary table 2.** Effect of speed on ataxic upper limb movements

|  | **Test** | **Mean/median** | **t** | **Z** | **p** |
| --- | --- | --- | --- | --- | --- |
| TS | FNT-S | 0.50 |  | -3.45 | <0.001 |
|  | FNT-F | 0.71 |  |  |  |
| SP-X | FNT-S | 5.16 | -4.23 |  | <0.001 |
|  | FNT-F | 7.79 |  |  |  |
| SP-Y | FNT-S | 3.43 |  | -3.58 | <0.001 |
|  | FNT-F | 5.38 |  |  |  |
| SP-Z | FNT-S | 3.95 | -4.63 |  | <0.001 |
|  | FNT-F | 6.01 |  |  |  |
| SP-aX | FNT-S | 108 | -4.38 |  | <0.001 |
|  | FNT-F | 170 |  |  |  |
| SP-aY | FNT-S | 155 | -4.08 |  | 0.001 |
|  | FNT-F | 211 |  |  |  |
| SP-aZ | FNT-S | 131 | -4.11 |  | 0.001 |
|  | FNT-F | 211 |  |  |  |
| RF-X | FNT-S | 1.50 |  | -2.62 | 0.009 |
|  | FNT-F | 2.00 |  |  |  |
| RF-Y | FNT-S | 1.78 | -1.75 |  | 0.098 |
|  | FNT-F | 1.98 |  |  |  |
| RF-Z | FNT-S | 1.78 |  | -1.63 | 0.103 |
|  | FNT-F | 2.30 |  |  |  |
| RF-aX | FNT-S | 1.69 |  | -0.22 | 0.825 |
|  | FNT-F | 1.49 |  |  |  |
| RF-aY | FNT-S | 1.39 |  | -2.14 | 0.033 |
|  | FNT-F | 1.70 |  |  |  |
| RF-aZ | FNT-S | 1.28 |  | -0.42 | 0.671 |
|  | FNT-F | 1.39 |  |  |  |
| Sta-X | FNT-S | 4.65 |  | -0.68 | 0.494 |
|  | FNT-F | 4.63 |  |  |  |
| Sta-Y | FNT-S | 3.73 |  | -1.33 | 0.184 |
|  | FNT-F | 4.71 |  |  |  |
| Sta-Z | FNT-S | 2.49 |  | -2.50 | 0.013 |
|  | FNT-F | 5.30 |  |  |  |
| Sta-aX | FNT-S | 4.06 |  | -0.36 | 0.717 |
|  | FNT-F | 4.18 |  |  |  |
| Sta-aY | FNT-S | 5.18 |  | -0.885 | 0.376 |
|  | FNT-F | 4.35 |  |  |  |
| Sta-aZ | FNT-S | 8.41 |  | -0.443 | 0.658 |
|  | FNT-F | 4.87 |  |  |  |
| SD-X | FNT-S | 0.23 | -4.04 |  | 0.001 |
|  | FNT-F | 0.34 |  |  |  |
| SD-Y | FNT-S | 0.15 | -5.13 |  | <0.001 |
|  | FNT-F | 0.22 |  |  |  |
| SD-Z | FNT-S | 0.17 | -4.75 |  | <0.001 |
|  | FNT-F | 0.25 |  |  |  |
| SD-aX | FNT-S | 4.78 | -4.44 |  | <0.001 |
|  | FNT-F | 7.19 |  |  |  |
| SD-aY | FNT-S | 7.64 | -3.09 |  | 0.006 |
|  | FNT-F | 10.7 |  |  |  |
| SD-aZ | FNT-S | 7.62 |  | -3.82 | <0.001 |
|  | FNT-F | 12.4 |  |  |  |
| cons | FNT-S | 0.10 |  | -1.00 | 0.314 |
|  | FNT-F | 0.10 |  |  |  |
| acc | FNT-S | 0.12 |  | -0.724 | 0.469 |
|  | FNT-F | 0.11 |  |  |  |
| CV of TS | FNT-S | 0.27 |  | -0.355 | 0.723 |
|  | FNT-F | 0.24 |  |  |  |

Paired samples *t*-test or Wilcoxon signed rank test was performed as appropriate. acc = tapping accuracy; aX = angular acceleration in X axis; aY = angular acceleration in Y axis; aZ = angular acceleration in Z axis; cons = standard deviation of time between taps; CV = median coefficient of variation, p = p-value; RF = resonant frequency; SD =standard deviation of amplitude; SP = power with 1-15Hz spectral range; Sta = stationarity of the signal; taps = number of taps registered by the touch screen;; t = *t*-test statistic; TS = tapping speed; Z = Wilcoxon signed rank test Z-score.

**Supplementary table 3.** Resonant frequency and stationarity in FNT

|  | | | FNT-S | | | FNT-F | | | |
| --- | --- | --- | --- | --- | --- | --- | --- | --- | --- |
| Variable | | | Mean/median | | p-value | Mean/median | | p-value | |
| RF-X | control | 1.69 | | 0.452 | | | 1.78 | | 0.421 |
|  | patient | 1.50 | |  | | | 2.00 | |  |
| RF-Y | control | 1.59 | | 0.537 | | | 1.98 | | 0.810 |
|  | patient | 1.78 | |  | | | 1.98 | |  |
| RF-Z | control | 2.28 | | 0.047 | | | 2.78 | | 0.052 |
|  | patient | 1.78 | |  | | | 2.30 | |  |
| RF-aX | control | 1.50 | | 0.405 | | | 1.78 | | 0.124 |
|  | patient | 1.69 | |  | | | 1.49 | |  |
| RF-aY | control | 1.60 | | 0.014 | | | 1.39 | | 0.019 |
|  | patient | 1.39 | |  | | | 1.70 | |  |
| RF-aZ | control | 1.49 | | 0.555 | | | 1.40 | | 0.936 |
|  | patient | 1.29 | |  | | | 1.39 | |  |
| Sta-X | control | 3.84 | | 0.592 | | | 19.03 | | 0.117 |
|  | patient | 4.65 | |  | | | 4.63 | |  |
| Sta-Y | control | 8.53 | | 0.044 | | | 3.32 | | 0.282 |
|  | patient | 3.73 | |  | | | 4.71 | |  |
| Sta-Z | control | 4.45 | | 0.039 | | | 18.31 | | 0.051 |
|  | patient | 2.49 | |  | | | 5.30 | |  |
| Sta-aX | control | 8.91 | | 0.065 | | | 9.35 | | 0.005 |
|  | patient | 4.06 | |  | | | 4.18 | |  |
| Sta-aY | control | 16.73 | | 0.117 | | | 21.86 | | 0.017 |
|  | patient | 5.18 | |  | | | 4.35 | |  |
| Sta-aZ | control | 8.41 | | 0.979 | | | 25.53 | | 0.003 |
|  | patient | 8.41 | |  | | | 4.87 | |  |

aX = angular acceleration in X axis; aY = angular acceleration in Y axis; aZ = angular acceleration in Z axis; RF = resonant frequency; Sta = stationarity of the signal. No significant differences were detected between patients and controls in stationarity or resonant frequency of upper limb movements.

**Supplementary table 4.** Correlation of FNT-S variables with SARA score.

| Variable | | Correlation coefficient |
| --- | --- | --- |
| taps | ρ | -0.376 |
|  | P | 0.112 |
| SP-X | r | -0.466 |
|  | P | 0.044 |
| SP-Y | ρ | -0.511* |
|  | P | 0.025 |
| SP-Z | r | -0.411 |
|  | P | 0.080 |
| SP-aX | ρ | -0.396 |
|  | P | 0.093 |
| SP-aY | r | -0.520* |
|  | P | 0.023 |
| SP-aZ | r | -0.605* |
|  | P | 0.006 |
| RF-X | ρ | 0.332 |
|  | P | 0.165 |
| RF-Y | ρ | -0.194 |
|  | P | 0.426 |
| RF-Z | ρ | -0.206 |
|  | P | 0.397 |
| RF-aX | ρ | -0.363 |
|  | P | 0.127 |
| RF-aY | ρ | 0.061 |
|  | P | 0.805 |
| RF-aZ | ρ | 0.077 |
|  | P | 0.753 |
| Sta-X | ρ | -0.055 |
|  | P | 0.822 |
| Sta-Y | ρ | -0.354 |
|  | P | 0.138 |
| Sta-Z | ρ | -0.374 |
|  | P | 0.115 |
| Sta-aX | ρ | 0.201 |
|  | P | 0.410 |
| Sta-aY | ρ | -0.109 |
|  | P | 0.657 |
| Sta-aZ | ρ | -0.631 |
|  | P | 0.004 |
| SD-X | r | -0.491 |
|  | P | 0.033 |
| SD-Y | r | -0.619* |
|  | P | 0.005 |
| SD-Z | r | -0.336 |
|  | P | 0.160 |
| SD-aX | r | -0.243 |
|  | P | 0.317 |
| SD-aY | ρ | -0.400 |
|  | P | 0.090 |
| SD-aZ | r | -0.685* |
|  | P | 0.001** |
| cons | ρ | 0.070 |
|  | P | 0.776 |
| acc | r | -0.164 |
|  | P | 0.502 |

acc = tapping accuracy; cons = standard deviation of time between taps; r = Pearson’s correlation coefficient; ρ =Spearman’s rank correlation coefficient; RF = resonant frequency; SD =standard deviation of amplitude; SP = power with 1-15Hz spectral range; Sta = stationarity of the signal; taps = number of taps registered by the touch screen; aX = angular acceleration in X axis; aY = angular acceleration in Y axis; aZ = angular acceleration in Z axis; * = significant correlation, **= significant p-value
